# Effective teaching of endoscopy: a qualitative study of the perceptions of gastroenterology fellows and attending gastroenterologists

**DOI:** 10.1093/gastro/gow003

**Published:** 2016-03-22

**Authors:** Daniel J. Zanchetti, Samuel A. Schueler, Brian C. Jacobson, Robert C. Lowe

**Affiliations:** Boston University Medical Center, Boston, MA, USA

**Keywords:** endoscopy curriculum, teaching endoscopy, gastroenterology fellowship training

## Abstract

**Background:** There is little information describing the perceptions of gastroenterology fellows and attending gastroenterologists of what constitutes effective teaching of endoscopy. We sought to identify common themes regarding endoscopy training methods and their impact among fellows and attendings.

**Methods:** Focus group exercises and surveys were conducted among fellows, about educational resources, teaching techniques and ways of improving the teaching of endoscopy. The fellows identified the ‘best' teachers of endoscopy, who were interviewed regarding their training in endoscopy, their teaching methods, key points of information, and opinions on endoscopy curriculum.

**Results:** Nineteen fellows (68%) had attended the American Society for Gastrointestinal Endoscopy First Year Fellows’ Endoscopy course and found it very helpful. Thirteen fellows(46%) had exposure to an endoscopy simulator, but their median duration of use was only 1 hour. Only two out of five fellowship programs used a formal endoscopic skill assessment tool and none of the programs had an endoscopy curriculum of which the fellows were aware. Fellows reported that they learned endoscopy best by performing procedures. They also volunteered that attending gastroenterologists used variable teaching methods, and might benefit from instruction on how to teach endoscopy. Ten attending gastroenterologists (77%) had received training in advanced procedures; none received formal training on teaching endoscopy: they all felt that such training would be beneficial.

**Conclusions:** A standardized endoscopy curriculum may be beneficial to fellows, who prefer to learn endoscopy by performing procedures—but they want explicit and specific instruction. Both those attending and the fellows thought that formal instruction for attending gastroenterologists on how to teach endoscopy would be beneficial, indicating a role for a ‘teach-the-teacher' curriculum.

## Introduction

Throughout gastroenterology fellowship training, fellows are exposed to a variety of different attending physicians who observe and direct them as they manipulate the endoscope [1–9]; however, the actual process of teaching endoscopy, is not well defined. There is currently no standardized or systematic approach to producing proficient and competent endoscopists. Whilst there are suggested threshold numbers of procedures that must be performed—as well as assessment and evaluation tools—a more systematic approach to the fundamental teaching of endoscopy is lacking; likewise, there are no available documents or videos to guide instructors in how to teach endoscopy. As the Accreditation Council for Graduate Medical Education (ACGME) moves towards a milestone-based approach within each specialty, this will also be applied to endoscopy training [10]. The American Society for Gastrointestinal Endoscopy (ASGE) and ACGME have jointly developed an objective-based approach to endoscopy training which takes into account the following three domains: (i) technical (psychomotor); (ii) cognitive; and (iii) integrative [6]. At the completion of fellowship, fellows should be capable of independently performing routine endoscopic procedures, including specific therapeutic maneuvers (e.g. polypectomy, hemostasis techniques, etc.) [8]. Although this milestone-based approach provides some guidance for training programs, our study aims to provide a better understanding of how a supervising faculty should teach endoscopy.

This study examined how fellows are taught endoscopy throughout fellowship and the degree of variability in the methods of teaching endoscopy that exists within and between different training programs. We also attempted to identify the methods of teaching endoscopy that are considered to be effective by both fellows and attending gastroenterologists, with the objective of providing this data to instructors and trainees in the discipline; furthermore, we hope to inform further investigation in this area, as well as potential interventions such as a “teach the teacher” curriculum, to improve the teaching of endoscopy.

## Methods

We conducted studies with focus groups of current gastroenterology fellows involved in fellowship programs at several centers in the Boston area, including Boston Medical Center, Tufts Medical Center, Brigham and Women's Hospital, Lahey Clinic and the University of Massachusetts Medical Center. Permission to include fellows in the studies was granted by the gastroenterology program directors at these institutions. Informed consent for the surveys and focus groups was obtained from all participating fellows. The focus group sessions (45–60 minutes in duration) included identical closed- and open-ended questions covering how the fellows learned endoscopy and their perceived needs for additional curriculum (Appendix 1). The fellows were also asked to identify two to three ‘best' teachers of endoscopy at their respective institutions.

In addition, the participating fellows were asked to complete a survey assessing their year of training, number of procedures performed, exposure to alternative methods in the teaching of endoscopy (e.g. the ASGE First Year Fellows’ course or simulators) and their exposure to assessment tools for upper endoscopy and colonoscopy (Appendix 2).

The attending gastroenterologists identified by the fellows as the best teachers were then interviewed separately by telephone. Informed consent was obtained. The interviews included closed- and open-ended questions identical to those posed to the fellows. Participants were asked a series of questions about their training, teaching styles and overall opinions on the teaching of endoscopy (Appendix 3). All the fellows’ focus group sessions and attending interviews were recorded, transcribed and coded by the investigators. Using a grounded theory approach, a thematic analysis was conducted to define those teaching methods that the attending gastroenterologists identified as being most helpful. We utilized member checking and peer debriefing techniques to increase the validity of our findings. NVivo software (QSR International Pty Ltd.) was utilized to assist with the analysis. This study was approved by the Boston University Institutional Review Board.

## Results

Overall, nine first-year, eight second-year, and eleven third-year fellows participated in this study (28 of 48 possible fellows: 58% participation rate). Each completed the surveys. The survey data from the fellows revealed that 19 fellows (68%) had attended the ASGE First Year Fellows’ course and found it very helpful (median score 5;interquartile range [IQR] 4–5 on a Likert scale, with 1 = very unhelpful and 5 = very helpful). Thirteen fellows(46%) had exposure to an endoscopy simulator at their institution; however, the median time spent with the simulator was one hour (IQR 1–3). Only two of the five fellowship programs used a formal endoscopic skill assessment tool and none of the programs had an endoscopy curriculum of which the fellows were aware.

The focus groups revealed that the fellows consistently reported learning endoscopy best by performing procedures (as opposed to classroom sessions, videos, or observing procedures). They identified procedure-related goals they had set for themselves, such as getting past the pylorus or achieving unassisted cecal intubation. Whilst some fellows felt that such goals were obvious, others wanted attending gastroenterologists to be more explicit in identifying goals for them to achieve, based on their levels of competency. Fellows appreciated specific and explicit instruction, and identified instances where a lack of instruction had led to frustration or delay in developing or mastering certain techniques and concepts. They did not think that they learned differently based on their year of training, although second- and third-year fellows reported a shift in their milestones towards more advanced techniques such as polypectomy. They noted that different attending gastroenterologists had variable teaching methods or styles (Table 1), which was at times perceived as beneficial to the learning process, whereas at other times it was felt to be a manifestation of the teacher’s limitations as an endoscopist and/or instructor. They were in agreement that there should be an endoscopy curriculum, although opinions varied on how to accomplish this, as some advocated for a more standardized curriculum with specific learning objectives while others felt that each fellow should learn at their own pace. Some wanted more dedicated endoscopy time, while others favored more use of simulators. Finally, fellows thought that their instructors would benefit from instruction on how to teach endoscopy.

**Table 1.** Focus group themes among gastroenterology fellows

**Fellows focus group themes identified**	**Representative examples (quotes from fellows focus groups)**
Learn best by performing endoscopy (*vs*. observation, didactics)	“I learn by actually doing it…when you first start there are 10000 things you're trying to remember. For them to go through the whole thing and for you to just watch doesn't help. You have to do it.” “I think just repetitive, muscle memory, and doing it over and over again.” “…learning colonoscopy just takes a lot of procedures at the end of the day.”
Self-identified procedure-related goals and milestones (e.g. cecal intubation)	“…inherently everybody will set their own milestones…it would be helpful if attending gastroenterologists let us know that at six months, we should be able to reach these milestones…” “As far as initially learning upper endoscopy…I learned in terms of milestones: intubating the esophagus…getting past the pylorus…getting into D2…I think it’s a very step-wise process.”
Graduated learning and independence	“Now when I do my colonoscopy… I'm not just trying to find the lumen… I'm actually looking for flat polyps…”
Diversified training from attending gastroenterologists (variably beneficial)	“Lots of variability between the attending gastroenterologists and for the most part, I think it's on them; it's their limitations and inability to explain themselves better.” “…getting past the sigmoid, or the various flexures…they all require different maneuvers and different techniques. I think [I learned] by just having different attending gastroenterologists gives you different points on how to get into those.”
Desire for more endoscopy curriculum	“I think putting in milestones and having evaluations every few months is at least a start in the right direction.” “I think it would be helpful to have a standardized curriculum…but everyone learns endoscopy at a different rate…it would have to be flexible.”
Attending gastroenterologists would benefit from instruction on teaching	“…having a curriculum designed for attending gastroenterologists to say 'hey, these are pearls of knowledge that you should be giving first years; these are key points you should be giving second years', and making that standardized.” “…they didn’t learn in a structured way. They don’t know how to teach in a structured way. They need help to structure what they’re saying and give more targeted feedback.”

Certain teaching techniques were noted by the fellows as being less helpful (Table 2); for example, they generally thought that two persons performing endoscopy—during which the attending gastroenterologist partially controls the endoscope—was less helpful than the fellow completely controlling the endoscope with the experienced endoscopist intently focused on guidance and technical assistance. Furthermore, fellows identified certain characteristics of attending gastroenterologists and/or the learning environment that were thought to be less helpful.

**Table 2.** Less-helpful teaching techniques identified by gastroenterology fellows

**Less-helpful teaching techniques and environments**	**Representative examples (quotes from fellows focus groups)**
Two persons performing endoscopy	“…if you're not torquing with one hand and dialing with the other then you're not really getting any experience.”
Losing the scope	“It would be nice if they just get through that really hard part but then let me try the rest.” “…the attending gastroenterologist just took over and said that I had done something wrong and then basically starting doing things on his own. I didn't feel that I learned very much from the complication.” “…It's frustrating when somebody takes the scope and doesn't explain what they're doing differently.”
Tense learning climate	“One very important thing with the attending is… how calm they are during the procedure… If I can tell they’re very anxious even before I start that makes me really anxious to start.”
Generic instruction	“General comments like 'scope the patient' or 'go' or 'go forward‘ aren't very useful when you're learning…”

Fellows consistently agreed on the most effective teachers of endoscopy in their programs and were able to identify three attending gastroenterologists considered “best teachers” at each institution.

These attending gastroenterologists comprised one clinical instructor, eight assistant professors, two associate professors, and two full professors, with 9 of13 (69%) performing advanced endoscopic procedures and 10 of13 (77%) reporting having received training in advanced procedures. Common themes from how attending physicians described their teaching styles, included maintaining a positive learning atmosphere identifying specific goals for the fellows during procedures, teaching to the level of the student, and positioning themselves next to the fellow during endoscopy (Table 3).

**Table 3.** Teaching themes identified by 'best teacher'attending gastroenterologists

**Teaching style themes from** **'best teacher'attending gastroenterologists**	**Representative examples (quotes from attending gastroenterologist interviews)**
Prioritizing safety of patients	“Know your limitations”
Creating a positive learning climate	“…for the patient's sake, and also for the fellow's learning, you've got to keep it positive. Be calm. Be patient. No yelling.” “…while patience is important, I think being able to fake patience is also just as important. If you can’t be patient, at least pretend you are.”
Teaching to the level of the learner	“I would never ask a first-year who is trying to learn how to advance a scope 'So, what is the differential that you're thinking about here?', I just wouldn't do that to them because I just think their brain is just too busy and should be focusing on the technical [aspects].“
Setting goals and milestones for fellows	“Early on in the first year, you try to establish a concept… for example, keep a straight scope…and then demonstrating or teaching how you do that. So, keep a straight scope, means pull back and reduce, minimize air, for example.“
Explicit communication for troubleshooting	“The reason I took it [the scope] wasn't because you were doing a bad job, it was that a patient was screaming, so it wasn't a good situation”; “If I find a fellow is having a problem and not progressing and I have to help them, I will do it, but I will explain what I'm doing, or how I'm doing it, etc.”
Standing next to the fellow	“I'm right next to the fellow where I can see their hand movements and their dial work … I can anticipate their moves and when their having trouble … if they're trying to get through the sigmoid or it’s a difficult corner, I will try to imagine what I would do in that situation.”

Notably, none of the attending gastroenterologists in our study were provided with any formal instruction on how to teach endoscopy, and a majority of these felt that formal teaching would have been helpful during their training. Similarly to the fellows, many of the attending gastroenterologists stressed that an endoscopy curriculum should be flexible, to allow for different styles; furthermore, most attending gastroenterologists felt that if they were to receive instruction on how to better teach endoscopy presently, it would be helpful to them. Comparisons were drawn to the move to improve detection rates for adenomas and that it would be fair to extrapolate more generally that attending gastroenterologists should also receive instruction on other parts of their practice, including how to more effectively teach endoscopy to trainees. Others observed that, while performing endoscopy becomes second nature, this is not necessarily true for effectively explaining or teaching the subject, and that instruction in this skill along with a formal endoscopy curriculum would be helpful.

## Discussion

Our findings suggest that gastroenterology fellows are interested in a more detailed endoscopy curriculum. A majority stated that the best way to learn endoscopy is by actually performing procedures. Fellows consistently expressed interest in their instructors being more explicit and specific during endoscopy procedures. Along these lines, we also found that both attending gastroenterologists and fellows indicated that instruction on how to effectively teach endoscopy may be beneficial to those giving training. It was noted by fellows that attending gastroenterologists did not necessarily learn endoscopy in a structured way and therefore may not teach it as such; furthermore, attending gastroenterologists noted that while the practice of endoscopy becomes second nature, the explanation of endoscopy to the learner is not necessarily as intuitive.

Given these findings, we believe there is a role for a teach-the-teacher curriculum. This curriculum might be derived from breaking down endoscopic procedures into key portions and skills and then having experts offer tips and detailed explanation on approach and technique. From our interviews with attending gastroenterologists identified by their fellows as “best teachers,” we have compiled a table of key information about the teaching of endoscopy which may be useful teaching tools for other gastroenterologists (Table 4). Given the limited number of endoscopists interviewed for this study, the fact that a number of key items of training information were extracted from our transcripts suggests that there is a wealth of endoscopy techniques that are used on a local level at programs throughout the country, which could become a key part of a national teaching curriculum in endoscopy.

**Table 4.** **The teaching of key informationin about endoscopy**

Navigate the sigmoid colon like climbing a spiral staircase (use rotary movements).
Use the Bolster Technique (applying abdominal pressure during upper endoscopy) to visualize Schatzki rings.^11^
Keep a short scope to stay off the esophageal or intestinal wall.
If the scope is taken from fellow, provide an explanation why, and try to return the scope when the impediment is resolved.
Navigate the transverse colon like playing a trombone (repeated insertions and withdrawals).
Use the pinkie finger to hold the flexible portion of the colonosope at the entry point, stabilizing position (the Pinkie Maneuver).^12^
When taking esophageal biopsies, open the forceps in the stomach and then withdraw towards the lesion.
Air is your enemy going in, but air is your friend going out.
Focus on one take-home lesson for fellow for each endoscopy case or session.
Do not go into a puddle: suction from above and keep your view.
Going around the hepatic flexure is like climbing a ladder… keep the rungs of the ladder in view at all times and keep yourself off the mucosa as you climb your way up.

We acknowledge the limitations inherent in our study, including that qualitative research precludes testing of hypotheses. Nonetheless, we consider our findings an important first step towards better understanding how trainees perceive instruction in the practice of endoscopy. We also found that those attending gastroenterologists deemed to be “best teachers” shared similar opinions about which methods work and which should be avoided. Another limitation of our study was lack of 100% participation by every fellow at each participating institution. Because our study included only programs in the Boston area, our findings may not be generally applicable; however, our findings can easily be used to initiate a conversation within other gastroenterology programs that may provide other important insights. As the ACGME moves to a milestone-based means of assessing trainees' progress, we think our findings provide a useful framework for considering the manner in which endoscopy is taught.

In conclusion, a standardized endoscopy curriculum with procedural goals may be beneficial to fellows, who prefer to learn endoscopy by performing actual procedures and benefit from receiving explicit and specific instruction from attending gastroenterologists. Attendings and fellows agreed that formal instruction for attending gastroenterologists on how to teach endoscopy would be helpful, yet none of the experienced endoscopists in our study reported receiving specific instruction on how to teach endoscopy, which may indicate a role for a “teach-the-teacher” curriculum. The teaching of 'pearls of wisdom' in endoscopy that we identified, suggest there is, in practice, variability in the teaching of endoscopy techniques which may benefit both instructors and trainees and be a valuable component of endoscopy curriculum.

## Appendix 1

**Table:**

**Focus group questions for gastroenterology fellows**
1) How do you think you learned upper endoscopy and colonoscopy?
2) What were important teaching tools you learned as a first year fellow?
3) Did you learn endoscopy differently as a second year and third year? If so, how?
4) What were some of the least helpful teaching experiences that you experienced as a fellow?
5) Do you think your education was consistent amongst attending physicians? If not, how variable was it?
6) What had the greatest impact on your endoscopy education?
7) Did the attending that you scoped with influence your endoscopy skills/learning curve? If so, how much?
8) Are there pearls of wisdom that you have taken away from your endoscopy education that you think all fellows should know or be aware of?
9) Do you think there should be a more standardized endoscopy curriculum? As an example, a curriculum that is milestone based with clear goals that fellows should/need to achieve at various times throughout fellowship (e.g. intubating the esophagus within the first 15 procedures).
10) How would you design an endoscopy curriculum if you were to create one?
11) Who would you identify as the best 2–3 endoscopy teachers?

### Appendix 2

**Table:**

**Survey questions for gastroenterology fellows**
1) What is your level of training?
2) Did you perform any upper endoscopy or colonoscopy prior to entering gastroenterology fellowship? If so, please comment.
3) Did you attend the ASGE first year fellow endoscopy course? If yes, how helpful was it?
4) Have you worked with a simulator as part of your endoscopy education? If so, approximately how often?
5) Aside from simulators or actually performing endoscopy and colonoscopy, what other audiovisual aids were used in your training (i.e. PowerPoint, textbooks, videos, etc.)?
6) How many upper endoscopies have you performed or did you perform as a first-year fellow?
7) How many upper endoscopies have you performed or did you perform as a second-year fellow (if applicable)?
8) How many upper endoscopies have you performed or did you perform as a third-year fellow (if applicable)?
9) How many colonoscopies have you performed or did you perform as a first-year fellow?
10) How many colonoscopies have you performed or did you perform as a second year fellow (if applicable)?
11) How many colonoscopies have you performed or did you perform as a third-year fellow (if applicable)?
12) Does your training institution use an assessment tool for colonoscopy?
13) Does your training institution use an assessment tool for colonoscopy?
14) How satisfied are you with your endoscopy education? (Answer choices: 1 = very satisfied; 2 = satisfied; 3 = neutral; 4=dissatisfied; 5 = very dissatisfied).

### Appendix 3

**Table:**

**Questions asked of attending gastroenterologists**
1) How many years out of fellowship are you?
2) What is your academic title?
3) What particular career track are you on within your institution?
4) Do you perform EUS/ERCP or are you trained in EUS/ERCP?
5) How would you describe your teaching style for teaching endoscopy?
6) How did you develop this style?
7) Did you have any formal teaching/training on how to teach endoscopy?
8) Do you teach endoscopy differently to a 1^st^ year, 2^nd^ year, or3^rd^ year? If so, how?
9) What do you think are important attributes to teaching endoscopy? Is there one you would select as most important?
10) Are there any key items of information that you offer to the fellows with regards to learning endoscopy?
11) Are there any key items of information that you would offer to faculty teaching endoscopy?
12) Do you think there should be a more standardized endoscopy curriculum? (As an example, a curriculum that is milestone based with clear goals of what fellows should/need to achieve at various times throughout fellowship, i.e. intubating the esophagus within the first 15 procedures)
13) How would you design an endoscopy curriculum if you were to create one?
